# Small ORFs, Big Insights: *Drosophila* as a Model to Unraveling Microprotein Functions

**DOI:** 10.3390/cells13191645

**Published:** 2024-10-02

**Authors:** Hélène Chanut-Delalande, Jennifer Zanet

**Affiliations:** Unité de Biologie Moléculaire, Cellulaire et du Développement (MCD), UMR 5077, Centre de Biologie Intégrative (CBI), CNRS, UPS, Université de Toulouse, 31062 Toulouse, France; helene.chanut@univ-tlse3.fr

**Keywords:** small ORF, smORF, peptides, microproteins, *pri*, *tal*, *Drosophila*, development

## Abstract

Recently developed experimental and computational approaches to identify putative coding small ORFs (smORFs) in genomes have revealed thousands of smORFs localized within coding and non-coding RNAs. They can be translated into smORF peptides or microproteins, which are defined as less than 100 amino acids in length. The identification of such a large number of potential biological regulators represents a major challenge, notably for elucidating the in vivo functions of these microproteins. Since the emergence of this field, *Drosophila* has proved to be a valuable model for studying the biological functions of microproteins in vivo. In this review, we outline how the smORF field emerged and the nomenclature used in this domain. We summarize the technical challenges associated with identifying putative coding smORFs in the genome and the relevant translated microproteins. Finally, recent findings on one of the best studied smORF peptides, Pri, and other microproteins studied so far in *Drosophila* are described. These studies highlight the diverse roles that microproteins can fulfil in the regulation of various molecular targets involved in distinct cellular processes during animal development and physiology. Given the recent emergence of the microprotein field and the associated discoveries, the microproteome represents an exquisite source of potentially bioactive molecules, whose in vivo biological functions can be explored in the *Drosophila* model.

## 1. What Are smORFs and Microproteins?

When genomes were sequenced, specific criteria were established to accurately annotate genes. To prevent the annotation of millions of Open Reading Frames (ORFs), i.e., DNA sequences starting with an ATG and ending with a stop codon, an arbitrary lower limit of 100 codons was set to define an ORF [1,2,3]. As a result, most small ORF (smORFs), also known as short ORFs (sORFs) or non-canonical ORFs (ncORFs), that do not exceed 100 codons, were not annotated [4]. However, the discovery in the *Drosophila* genome of small ORFs, named *pri*, which were initially annotated as non-coding, are capable of being translated into peptides only 11 amino acids (aa) long, and have crucial developmental roles [5,6,7], has entirely blurred the frontier between coding and non-coding genomes. This revealed that other as-yet-unannotated smORFs could also be coding and translated, thus pioneering the field of microproteins. Since then, the function of several microproteins has been studied in various organisms, including *Drosophila*, plants, yeast, bacteria, and human cells [8,9,10,11,12,13,14,15]. Microproteins refer to polypeptides translated from small ORFs and come with different names in the literature: micropeptides, microproteins, smORF peptides, SEPs (small ORF encoded peptides), AltORF (Alternative ORF), and small proteins. In addition, the small ORF itself is called ncORF for non-canonical ORF, as opposed to the canonical ORF defined as having a size greater than 100 codons, present in the mRNA and coding for a so-called canonical protein. As the terminology distinguishing a peptide from a protein is based on its length, with the threshold of 50 aa, we now term any product translated from a smORF (defined as less than 100 codons) a microprotein, which thus includes all polypeptides shorter than 100 aa. Therefore, smORF refers to the DNA/RNA nucleotide sequence, and microprotein to the truly translated polypeptide. This new family of microproteins is now known as the microproteome, smORFome, or non-canonical proteome.

## 2. How to Identify smORFs and Microproteins?

**Figure 1:**
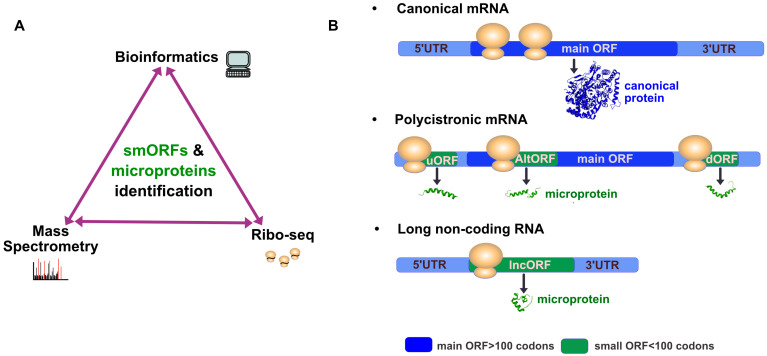
Methods of small ORF identification and their localization in RNA molecules. (**A**) The putative smORFs present in the genome can be either predicted by dedicated bioinformatic pipelines or identified by Ribo-seq data analysis. Improvements in mass spectrometry approaches allow the identification of novel microproteins. Combining these methodologies enables the identification of smORFs and the microproteins potentially encoded in the genome. (**B**) The mRNA molecule was classically viewed as monocistronic and encoding one canonical protein from its main ORF (in blue). SmORFs, defined as being under 100 codons (in green), are mainly found in coding RNAs (mRNA), which makes them polycistronic, and non-coding RNAs such as long non-coding RNA (lncORF). In mRNA, smORFs can be nested in the 5′UTR (uORF) and 3′UTR (dORF), and within the main ORF in an alternative frame (AltORF).

Different approaches have been developed to identify, from millions of potential smORFs, the ones having a true coding potential (Figure 1A). Among them, adapted computational approaches have been crucial to predict putative coding smORFs. The first bioinformatic analyses estimated the existence of several hundreds of smORFs in the *Drosophila* genome [16,17]. However, recently, computational methods progressively integrating machine learning improved their prediction. For instance, MiPepid detects smORFs in the human genome following training on a dataset of high confidence of already proved coding smORF sequences [18], and DeepCPP recognizes nucleotide patterns around start codons, named nucleotide bias, to detect smORF in vertebrate and invertebrate genomes [19]. However, these methods predict different numbers and sets of microproteins, and therefore need further experimental validation.

To overcome the limitations of the in silico approach and possibly the resulting false-positive prediction of microproteins, an experimental approach, i.e., ribosome profiling (Ribo-seq), can be used to detect precisely the actively translated RNA sequences [20]. This technique has revolutionized the field of microproteins, providing experimental proof of smORF translation. This method consists of isolating and sequencing Ribosome-Protected RNA Fragments (RPFs), corresponding to RNA sequences in the course of translation, from any biological sample, thus providing an exhaustive view of the translational landscape [20,21,22]. This approach unveiled that smORFs are not only present in some non-coding RNAs (ncRNAs), but also in some mRNAs, mostly in 5′UTR but also in 3′UTR and within the main—or canonical—ORF in an alternative frame [23,24] (Figure 1B). SmORFs have thus been classified and named according to the type of RNA molecules on which they are localized (coding or non-coding RNA) and their localization on the mRNA molecules (Figure 1) [25]. Upstream ORFs (uORFs), present in the 5′UTR, are found in most eucaryotic mRNAs and regulate the translation of the main downstream ORF, through their RNA sequence itself, which can retain ribosomes to control their access to the main ORF [26]. Thus, uORFs can be translated and represent an important source of microproteins [27,28]. Functional studies on some of them have shown that they can interact with and regulate canonical proteins, either translated from the main ORF found downstream of the uORF [29] or with another canonical protein encoded by a different mRNA [28,30]. Although experimental evidences showed that smORFs present in the 3′UTR (dORFs) can be translated into microproteins, functional data are still scarce, so their role is not yet well understood [27,31]. Besides uORF, lncORF microproteins encoded from long non-coding RNAs (lncRNAs) are a major source of microproteins since 30% of transcribed lncRNAs appear to be translated in human cells and in *Drosophila* [32,33]. Notably, microproteins can also be translated from smORFs present in circular RNAs [34] or pre-miRNAs [35], highlighting that smORFs are hidden throughout the genome. To complicate things a little further, Ribo-seq method revealed non-AUG translation start sites when combined with different drugs (harringtonine or lactimidomycin) that block and accumulate ribosomes at initiation sites [27], thus substantially increasing the repertoire of putative microproteins. Finally, Ribo-seq reveals that translation is pervasive and increases with aging, carcinogenesis, or neurodegeneration [36,37,38]. However, physiological consequences of the translation landscape reshaping and the functions of resulting microproteins still remain unknown. Collectively, Ribo-seq data, mainly from mammalian cells, uncover the existence of thousands of microproteins whose functions are largely elusive.

In *Drosophila melanogaster*, Ribo-seq experiments dedicated to the identification of smORFs have been adapted to sequence RPFs only from the polysomal fraction (Poly-Ribo-seq), i.e., the fraction containing RNA molecules bound to at least two ribosomes, providing evidence of their translation [32,39,40,41].

Although Ribo-seq appears to be a powerful tool for discovering smORFs, these experiments have limitations. Indeed, the experimental protocol, which is not trivial to set up, requires further improvements for each type of sample and organism to obtain qualitative data [42]. Sequencing depth must be very high, making experimentation expensive, and bioinformatics analyses require the use of different Ribo-seq analysis tools to reliably identify smORFs, which are actually translated [43]. Despite the prominent impact of Ribo-seq on the field of smORF research, the evidence it provides for translation does not prove that a microprotein is produced.

Mass spectrometry (M/S) is the gold standard method for confirming the existence of microproteins. However, due to their low level of expression and their small size, the detection of a large proportion of translated peptides remains difficult [44]. A peptidomic-based strategy thus appears more adapted to the discovery of microproteins since, unlike proteomics, this approach does not require enzymatic digestion and allows the detection of endogenous peptides. Dedicated methods for the extraction and the enrichment of small proteins from samples, combined with the use of a state-of-the-art mass spectrometer and adapted bioinformatics workflows, largely contribute to the identification of novel microproteins [45]. Indeed, the combination of these experimental methods with a custom database, whose peptide sequences are predicted from transcriptomic and Ribo-seq data, allowed the discovery of unannotated microproteins in *Drosophila* [45,46]. Recent peptidomic studies in *Drosophila*, human cell lines, or mice have led to the discovery of several hundred microproteins, which may constitute a set of candidates to be further functionally tested in vivo [8,27,46,47,48]. 

To date, the exhaustive number of microproteins in any organisms, whose estimation varies from several hundreds to several thousands, is highly speculative and appears to depend on the method of identification, physiological conditions, and stages of development [33]. Several databases, such as OpentProt [49], SmProt [50], MetamORF [51], and sORFs.org [52], use published experimental and computational data to constitute a bank of predicted microproteins, whose estimated number is highly variable. For instance, SmProt predicts tens of thousands microproteins encoded by the human or the *Drosophila* genomes [50,53]. Recent experimental data and computational analyses allowed the annotation of more than 1000 microproteins in the *Drosophila melanogaster* genome [54]. A large number of studies on human cells or tissues using Ribo-seq and mass spectrometry analyses enabled the annotation of roughly 7000 microproteins in the human genome [25].

The progress of methods to detect microproteins and the attractiveness of this new field should lead to a growing number of confirmed translated microproteins in all organisms. This prospect is incredibly exciting, given the seemingly infinite potential for bioactive peptide discovery [55,56]. Since microproteins have been found recently to play a major role in cancer development [30,57,58,59], they actually benefit from a particular attention in oncogenesis research due to their potential as therapeutic agents or targets for cancer treatment. However, understanding the function of all these microproteins constitutes a bottleneck and different strategies are implemented to meet this challenge. In the following chapter, we will focus on studies carried out in *Drosophila* to illustrate the functions of the microproteins in vivo that have been studied to date.

## 3. Functions of Microproteins in *Drosophila*

The Pri (Polished rice) peptide, also known as Tal (Tarsal-less) [6,7] or Mlpt (Mille-pattes) in *Tribolium* [5], strongly contributed to the emergence of the microprotein field, demonstrating that smORFs as short as 36 nucleotides could be translated. Pri has been the most extensively studied microprotein, not only in *Drosophila* but also in other arthropods. In this review, we will first depict the various functions of Pri in *Drosophila* and other arthropods and then report on the roles of other microproteins in *Drosophila*.

### 3.1. *pri* Peptides as the Pioneers of the Microprotein Field

The *pri* gene was initially identified due to its remarkable dynamic expression pattern during embryonic development [60] and the strong developmental defects provoked by its genetic deletion [6,7]. Pri peptides, 11–32 aa long, are produced from a 1.5 kb long polycistronic mRNA containing four smORFs. The four Pri peptides share the LDPTGQY sequence with no predicted structure, which is conserved among insects and found in crustacean species [6,7]. Hemipteran species additionally contain a 80 aa long peptide, comprising a conserved sequence of 14 aa, but different from LDPTGQY, and whose role is not yet understood [61].

**Figure 2:**
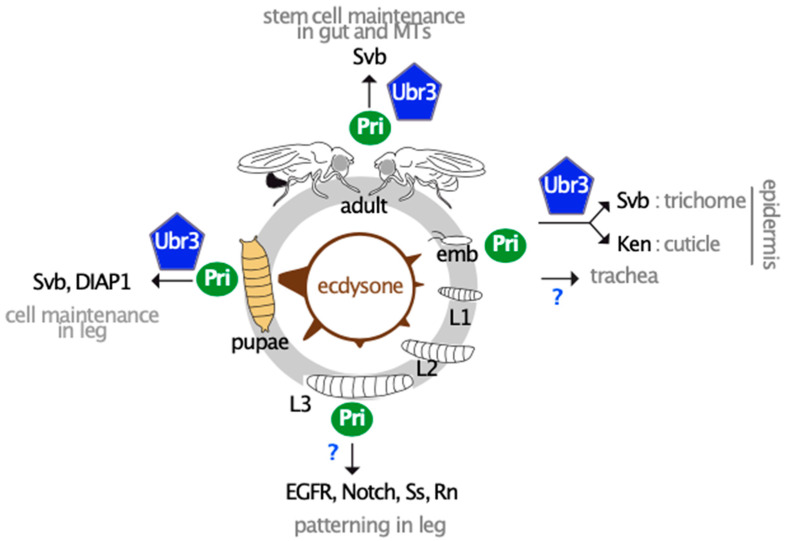
Pri peptides play pleiotropic roles throughout *Drosophila* development. Pri peptides mediate the function of ecdysone throughout the *Drosophila* life cycle to control the timing of morphogenetic processes in diverse tissues but also to protect cells from apoptosis. They execute several functions simultaneously in the same tissue (as shown in embryonic epidermis) but also in two or more tissues at the same time (in epidermis and trachea during embryogenesis). Understanding the molecular functions of Pri peptides allows the comprehension of how they fulfill their pleiotropic roles. Multiple functions of Pri peptides require their partner Ubr3, and together they modify the activity of the Svb and Ken TFs but also DIAP1. However, studies on leg patterning indicate that Pri peptides induce changes in the activity of several signaling factors and TFs (EGFR, Notch, Spineless, and Rotund) independently of Ubr3, suggesting that additional functions remain to be elucidated.

The *pri* gene is highly expressed in specific tissues at developmental transitions (Figure 2), supposing that Pri peptides are produced in large quantities at specific time points. The small size of Pri peptides suggests their fast translation, which favors their rapid function following their expression. Together, these data led researchers to propose that Pri peptides are produced rapidly and in large amounts in a given tissue to trigger their function in a very specific spatiotemporal scale. In addition, genetic experiments performed in epidermal tissues showed a cell non-autonomous function of Pri peptides [7,62,63,64]. Given their small size, Pri peptides could diffuse passively to neighboring cells through cytoplasmic bridges [65,66]. Although the function of this diffusive property is not yet understood, it might serve to equally distribute peptides in the tissue allowing their synchronous role with adjacent cells.

#### 3.1.1. Pri Displays Pleiotropic Functions in Diverse Tissues at the Same Time

Various functions of *pri* have been described in insects revealing its pleiotropic roles throughout animal development. In *Tribolium*, *mlpt* has been initially defined as a segmentation gene [5]. In *Drosophila*, *pri* is expressed widely along development as exemplified by its very dynamic expression pattern during embryogenesis in the tracheal system, gut, and epidermis, and also in the adult leg and wing [6,7,67]. These tissues are strongly affected when *pri* function is deregulated, for example, epidermal cells deficient for *pri* function lack cytoplasmic apical structures called trichomes [6,7].

#### 3.1.2. Pri Peptides Belong to a Tripartite Pri/Ubr3/Svb Module Controlling Epidermal Differentiation

When the role of *pri* in trichome formation was discovered, the transcription factor (TF) Shavenbaby (Svb) was the most downstream player known to control this process [68]. Svb directly triggers the expression of effector genes that participate in the building and the maintenance of the trichome [69,70,71]. Deciphering the molecular interaction between Svb and Pri peptides in the control of trichome formation greatly contributed to the understanding of the molecular mode of action of Pri peptides [72,73]. The combination of in vivo analyses and biochemical experiments in *Drosophila* S2 cells (genome wide screen) brought a precise understanding of the molecular function of Pri peptides during epidermal differentiation [72,73]. Pri peptides interacts with the E3 ubiquitin ligase Ubr3, a large molecule which is ubiquitously expressed in the organism and triggers the ubiquitination of specific proteins to allow their degradation via the proteasome [74]. The binding of Pri peptides to Ubr3 modifies the repertoire of its targets and Ubr3 acquires the potential to recognize additional targets, including Svb [73]. The Svb TF is initially produced as a long protein that represses the transcription of its target genes. Its N-terminal part contains a degron domain, i.e., a sequence that address the protein to the proteasome. This degron is recognized by the Pri/Ubr3 complex and ubiquitinated by Ubr3. This post-translational modification leads to the partial degradation of Svb through the proteasome and the resulting processed form, which has lost its repressor domain, activates the transcription of effector genes [72,73]. Therefore, the Pri/Ubr3 complex controls the conversion of Svb from a repressor into an activator of transcription. Adult epidermal cells are similarly producing trichomes using the same molecular mechanism showing a reiteration of the Pri/Svb/Ubr3 module [73]. Overall, these molecular studies revealed how very small microproteins can modify the activity of a larger protein, here Ubr3, with ubiquitous roles, to enlarge the spectrum of its biological functions for specific roles during development [73].

The processing of Svb to trigger trichome formation induced through a two-step process raised the question of the role of such a sophisticated mechanism controlled by the Pri peptides. Understanding how *pri* expression is regulated brought a larger vision of the function of Pri peptides. Based on a genetic screen, the expression of *pri* was shown to be directly regulated by the steroid hormone ecdysone, which controls the timing of important developmental transitions and metamorphosis during insect development [63,75]. The receptor of ecdysone, EcR, directly binds to the genomic region of *pri* composed of a large number of enhancers spread over at least 50 kb, each of them carrying specific spatiotemporal activities [75]. Through its cis-regulatory sequences, *pri* integrates the systemic signal of ecdysone and converts it into specific responses in tissues. *pri* responds to ecdysone in a large variety of cell types, illustrating its function as a wide responsive gene of ecdysone [63,75,76]. While other responsive genes of ecdysone encode nuclear receptors regulating a temporal cascade of transcriptional networks, the Pri peptides offer an alternative mode of action of ecdysone in tissues, directly regulating the processes of differentiation. As Pri peptides modify the activity of a large ubiquitous enzyme, their mechanistic role might facilitate the acquisition of novel functions of ecdysone.

#### 3.1.3. The Functions of the Pri/Ubr3/Svb Module Are Conserved across Insect Species

The *pri* gene is present in the large phylum of arthropods [5,6] and functional studies on Pri peptides have now been carried out on a broad array of insect species, i.e., the coleoptera *Tribolium castaneum*, the hymenoptera *Nasonia vitripennis*, and three hemipteran species *Gerris buenoi*, *Oncopeltus fasciatus* [77], and *Rhodnius prolixus* [61]. These studies established that the roles of Pri peptides are conserved in epidermal derivatives and leg formation, but also in the segmentation of insects. These roles are shared with the E3 ubiquitin ligase Ubr3 and the TF Svb, highlighting the conservation of the functional Pri/Ubr3/Svb module and its roles in embryonic/postembryonic development of insects [77]. However, the *Drosophila melanogaster* dipteran species diverges from other species since the function of the Pri/Ubr3/Svb module in segmentation has been lost due to the restriction of *svb* expression in early *Drosophila* embryos [77]. A similar absence of *pri* function in segmentation has been described for another dipteran species, *Clogmia albipunctata*, which might also relate to *svb* expression changes [78].

#### 3.1.4. Pri Peptides Display Pleiotropic Functions Throughout Tissue Differentiation

Detailed analyses of the functional role of *pri* in the respiratory system, embryonic epidermis, and leg development sustained that Pri peptides play a variety of roles throughout tissue development, using different molecular partners, which are partially identified. In the respiratory system, *pri* is expressed throughout the formation of the tubular network [6,7]. In this organ, Pri peptides control both the fate decision of cells involved in the formation of specific branches and morphogenesis of tubes through actin cytoskeleton remodeling, and deposition of the extracellular matrix in the lumen of the tubes [7,76,79]. However, Pri molecular partners involved in the tracheal system have not yet been identified. In embryonic epidermis, *pri* plays an additional role to trichome formation since the cuticle, which is the exoskeleton that entirely covers the animal, is dramatically affected in the absence of *pri* [80]. Under the control of ecdysone, Pri peptides regulate the production of a proper embryonic cuticle by inhibiting the expression of a large group of cuticle genes, which are normally expressed at later stages [80]. This function is executed by Pri peptides and Ubr3, which together control the activity of the Ken transcription factor. Therefore, in embryonic epidermis, Pri peptides, with their Ubr3 partner, coordinate two differentiation programs governed by two distinct transcription factors, Svb and Ken, for proper epidermis function [80]. Finally, during leg development, *pri* is expressed at two specific time points in a spatially restricted manner corresponding to two main functions during leg patterning and later on for cell survival [64]. While Pri peptides act on multiple factors independently of Ubr3 during leg patterning at larval stage, i.e., tarsal transcriptional programs (Rotund and Spineless TFs) and Notch and EGFR signaling pathways [62,64], Pri peptides and Ubr3 trigger Svb processing to protect cells from apoptosis during metamorphosis [64]. Identifying Pri peptide partners other than Ubr3 would constitute an entry point to decipher novel Pri functions.

#### 3.1.5. Reiteration of the Pri/Ubr3/Svb Module Acting in Other Tissues

Studying the functions of *pri* or *svb* at post-embryonic stages in *Drosophila* showed that the Pri/Ubr3/Svb module is reused in other tissues than the epidermis and leg to control tissue integrity during adulthood. *svb* and *pri* genes are expressed in stem cells of the digestive system (Malpighian tubules and intestine) where the Svb protein is processed in its transcriptional activate form to protect stem cells from apoptosis during adulthood [81,82]. Interestingly, *svb*, but not *pri*, is also expressed in the enterocytes, the intestinal differentiated cells, where the repressive form of Svb is required to trigger and maintain cell differentiation [82]. Post-embryonic roles of *pri* have also been described in the kissing bug *Rhodnius prolixus*, an important vector of the *Trypanosoma cruzi* parasite, which causes the Chagas disease, showing its function in digestive physiology and molting [83].

In summary, since their discovery, the pioneer Pri peptides have been extensively studied for more than 15 years, which make them one of the best characterized microproteins. Together, these studies reveal that Pri peptides have multiple features, i.e., dynamic expression patterns, diverse molecular partners, and a large number of targets, collectively conferring pleiotropic functions throughout development and adult life.

### 3.2. Microproteome Is a Pool of Regulatory Molecules

Given the tremendous number of uncharacterized microproteins, functional screens have been conducted to investigate their biological functions. Screens in human cell lines, aiming at identifying microproteins involved in cell growth and defining their interactomes, revealed their involvement in diverse cellular processes, such as translational regulation and endocytosis [27,28].

In *Drosophila*, functional screens have been conducted to explore the roles of the annotated microproteins in vivo by inducing their loss of function through RNAi or CRISPR techniques [64,84]. The specific microprotein depletion in developing legs showed that a substantial proportion of the tested microproteins (23/93) regulates leg morphogenesis [64]. Observations of defects in tissue growth, cell survival, patterning, and cuticle formation suggest that microproteins play regulatory roles in diverse cellular processes that drive the distinct morphogenetic events essential for leg formation [64]. Using bioinformatic analyses, Bosch et al. identified 298 microproteins conserved between human and *Drosophila* amongst the 1000 annotated microproteins in the *Drosophila* genome [84]. The knock-out (KO) of 115 of those conserved genes by CRISPR/Cas9 revealed that 14 are essential genes. In the same study, KO mutant flies for 25 other microproteins that were never characterized in other organisms were assessed for viability under normal and stressful conditions (here food-induced metabolic stress). Nearly all KO flies were viable and fertile in normal conditions, but several KO flies exhibited phenotypes under stressful conditions such as developmental delays, low viability, or lethality [84]. In *Drosophila*, microprotein sequence analyses showed that microproteins are enriched in transmembrane domains and mitochondrial-associated peptide motifs [64,84], as also observed in other species, suggesting a role of microproteins in membrane biology and mitochondria metabolism [85,86]. Interestingly, the analysis of genes encoding mitochondrial microproteins revealed distinct dynamic expression patterns during embryogenesis, suggesting that microproteins contribute to the cell and tissue specificities of mitochondrial activity over time [84].

### 3.3. Microproteins and de novo Genes

De novo genes are new protein-coding genes that recently appeared and are specific to a clade or to a few species. They emerge from non-coding regions, as well as from lncRNAs, and can evolve rapidly [87]. Despite their young age and their lack of conservation, the new genes may be essential for *Drosophila* development [87,88]. Since microproteins might have emerged from lncRNAs through the acquisition of robust translation during evolution, they could represent an important source of de novo proteins [28,32,87,89]. Interestingly, among 555 de novo genes recently identified in *Drosophila melanogaster* [90], we observed that half of them (225) code for microproteins, showing that genes encoding for microproteins strongly contribute to evolution. It has been proposed that the acquisition of new coding gene status involves several sequential steps: initial transcription, followed by ribosome binding, translation, and finally, the sustained maintenance of robust translation [32]. The maintenance of this translation may be enhanced by the functional properties of the nascent microprotein, which improve the organism fitness [32]. The pervasive ribosome binding could represent a mechanism for the emergence of new protein products that could confer an adaptive advantage to the organism. Although no de novo microprotein has been investigated to date for its contribution to *Drosophila* fitness, several newly born genes have been shown to be essential for *Drosophila* [87,88], which may presage that some de novo microproteins may confer an advantage for animal well-being.

### 3.4. Microproteins Represent an Additional Layer of Regulators for Biological Processes and Protein Activity

**Figure 3:**
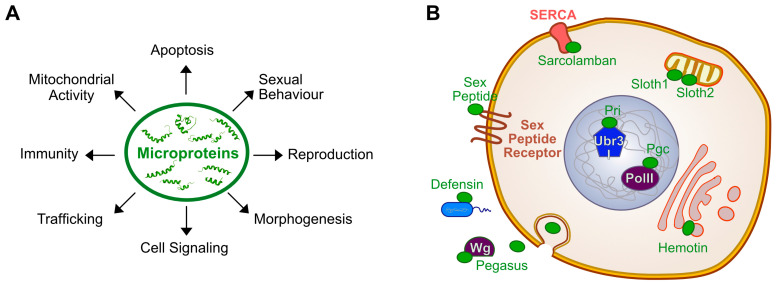
Microproteins regulate multiple physiological, developmental, and cellular functions in *Drosophila*. (**A**) Studies investigating the roles of microproteins in *Drosophila* show that they regulate a wide range of biological functions, as illustrated here. (**B**) Schematic representation of microproteins and their molecular targets identified so far in *Drosophila*. Microproteins regulate different types of canonical proteins localized in all cellular compartments, such as in nuclei with Pgc (Polar granule component) binding to Polymerase II and Pri peptides to Ubr3. Microproteins can also localize to membranes, like Hemotin in endosomes, or Sloth1/2 in mitochondrial membrane. They can also be secreted: Pegasus interacts with Wg (Wingless) in the extracellular space, Sex Peptide binds to its GPCR (G Protein-Coupled Receptors) receptor (Sex Peptide Receptor) and the AMP Defensin attaches to bacteria cell wall (in blue).

Since the first studies in *Drosophila* (reviewed in [91]), a significant number of microproteins has been described, revealing their various roles in vivo. Approaches to investigate microprotein functions rely on gain- and loss-of-function and phenotypic analyses, specifically examining the physiology and/or morphology of the tissue or organ in which the microprotein is expressed. The field greatly benefits from CRISPR/Cas9 genetic tools, which enable, for example, the generation of precise mutations at endogenous loci to disrupt smORF translation (codon start or smORF sequence deletion) or to tag the microprotein [80,92,93]. Finally, the molecular target of the microprotein can be identified by proteomic approaches. In this section, we review recent findings on the roles of microproteins in *Drosophila* development and physiology, illustrating their extensive functions across tissues over time (Figure 3).

In addition to Pri peptides and their essential roles in morphogenesis and cell survival during development, loss of function of other microproteins leads to developmental defects of varying degrees of severity. For instance, Pegasus, a 80 aa microprotein carrying a signal peptide, has been shown to increase lethality in *Drosophila* when deleted [94]. Its function has been studied in the wing imaginal disc, where it is expressed in the region that gives rise to the adult wing. Secreted Pegasus is essential for the diffusion of Wingless and the establishment of its gradient, which is necessary for patterning the number of mechanosensory bristles at the wing marge [94]. Another example is wurmchen 2 (wrm2), a 57 aa transmembrane microprotein, whose depletion results in larval lethality. Although it localizes to the apical extracellular matrix of the embryonic tracheal tubes, no morphogenetic defect was observed, suggesting its essential role in later developmental processes [93]. Notably, wrm2 is encoded by a bicistronic gene (*wurmchen1/2*), which transcribes an RNA molecule also encoding wurmchen 1, a small transmembrane protein of 162 aa, essential for the septate junction complex in the embryonic tracheal tube [93]. Unlike *pri* [6,7] or *sarcolamban* [12] genes, which are poly/bicistronic encoding microproteins with redundant biological functions, the *wurmchen1/2* gene encodes two microproteins localized within the same protein complex but displaying distinct biological roles [93]. Similarly, the two Sloth1 and Sloth2 microproteins, encoded by the bicistronic gene *sloth1/2*, are part of the same protein complex III in the mitochondrial inner membrane, but each of them has a unique role since they are not functionally redundant [92]. Sloth1 and Sloth2 are essential microproteins involved in mitochondrial activity in neurons [92]. Together, these studies support the existence of polycistronic genes in eukaryotes, as observed previously in human cells [23,27]. They are highly represented among smORF genes since 32 smORFs are encoded by polycistronic transcripts among the 298 smORFs conserved between *Drosophila* and human [84].

Another specific feature of microproteins is their significant enrichment in transmembrane motifs [85] and, in *Drosophila,* two microproteins were characterized as important regulators of cell trafficking [95,96]. Hemotin is a 88 aa transmembrane microprotein localized in the membrane of early endosomes in *Drosophila* macrophages, required for endosome biogenesis [95]. Its depletion results in abnormal, enlarged endosomes that are less efficient at degrading bacteria and pathogens, leading to a reduced resistance to infection and a diminished immunity. Noteworthy, the vertebrate homolog of Hemotin is the 88 aa microprotein Stannin, and both share a well-conserved transmembrane domain and molecular properties [95]. More recently, the microprotein Purriato was characterized as a transmembrane protein important for regulating autophagy in *Drosophila* muscles and thus preserving muscle fiber integrity [96]. These data support that microproteins are a valuable source of new players in membrane biology.

The family of Antimicrobial Peptides (AMPs) participates in the *Drosophila* innate immune system and is largely composed of microproteins since 23 of the 32 AMPs are less than 100 aa [97]. Their primary mode of action is to disrupt bacteria and fungi membranes through their insertion into the microbial membrane. Each AMP family appears to fight a specific group of pathogens, which is important for maintaining a balanced microbiome while effectively fighting infection. However, these AMPs appear to play other roles apart from their primary function in immunity. For example, the AMP Defensin, known to inhibit the growth of Gram-negative bacteria, induces tumor cell death [98]. Indeed, the presence of a tumor increases the expression of both Tumor Necrosis Factor (TNF) and Defensin. TNF alters the charge of tumor cell membranes, making them more sensitive to Defensin, which in turn induces cell death and tumor regression [98]. Thus, the function of microproteins could be co-opted in other biological processes, similarly to canonical proteins.

Finally, microproteins exhibit important roles in reproduction and sexual behavior. The iconic Sex Peptide (SP, 55 aa), discovered by biochemical approach before the birth of the microprotein field, is one of the best-characterized microprotein in *Drosophila* as SP is a crucial regulator of the Post-Mating Response in females [99]. Recently, the smallest microprotein MSAmiP (9 aa), found to be encoded by the *Drosophila* genome, is localized in the alleged *msa* lncRNA [100]. MSAmiP is expressed in the male accessory glands, which are the analog of the human prostate in *Drosophila*, and is required for sperm competition [100]. Furthermore, the translation of MSAmiP from the *msa* lncRNA, already characterized as a pre-miRNA, reveals the bimodal functions (non-coding and coding) of this gene. Another microprotein encoded from a previously annotated lncRNA is the microprotein IBIN (for Induced By INfection), first described as a lncRNA involved in immunity [101]. Surprisingly, IBIN is overexpressed in the eyes of flies after sighting a wasp and is required to accelerate mating behavior, thus coupling vision, changes in gene expression, and sexual behavior [102].

These recent findings have demonstrated that microproteins in *Drosophila* play crucial roles in a large variety of cellular and physiological processes across different cell types during development, highlighting their importance as a biological additional regulatory layer (Figure 3).

## 4. Conclusions

Since the discovery of the microprotein field, both experimental and computational biology have made significant advances leading to the identification of thousands of microproteins encoded by small Open Reading Frames (smORFs) in the genomes of all living organisms. Important recent contributions are coming from vertebrates studies, especially of humans [27,28,30,48,103,104]. Indeed, microproteins have been shown to play important roles in human diseases, especially in cancer where they act as tumor suppressors or oncogenic factors (for review, see [30,57]). Moreover, microproteins represent a source of cancer neoantigens, thus opening up great possibilities in terms of therapeutic approaches, e.g., as targets of vaccines [59]. Despite these important advances, only a small proportion of microproteins have been investigated for their biologic functions, particularly at the organismal level, indicating that numerous exciting discoveries remain to be made given the large number of microproteins whose functions are unknown. If we consider the possibility that many of the canonical proteins are regulated by one or more microproteins, as exemplified with the mammalian SERCA pump regulated by two microproteins [105], the elucidation of the functional repertoire of microproteins will require a substantial body of research. Large-scale approaches using proteomic, genetic, or computational methods, which proved their effectiveness [27,28,46,64,84,106,107], remain techniques of choice for undertaking functional studies of microproteins. Up to now, the evolutive conservation of microproteins constitutes an important requisite to define the importance of microproteins. However, investigations of the interactomes of young human microproteins reveal their ability to interact with essential canonical proteins via short amino acid motifs mediating protein–protein interactions, despite their lack of conservation [28]. This result suggests that sequence conservation is not a compulsory criterion of functionality since these novel microproteins, specific to the human clade, can interact with canonical and conserved proteins through these peptide motifs [28]. Instead, these data indicate that peptide motifs in de novo microproteins could mediate interactions with other proteins to modulate their activity. As illustrated by the Sarcolamban/Phospholamban microproteins regulating the SERCA muscle pump in vertebrates and invertebrates, non-conserved microproteins from one species could interact with canonical proteins from other species, which, despite their lack of sequence conservation, can be functionally swapped due to their conservation in the 3D structure [12]. Altogether, the use of the *Drosophila* model has proven to be highly relevant for elucidating the mechanisms regulating human physiology and pathology, thus highlighting the importance of performing functional studies of microproteins in this species [108]. Finally, the original and eclectic roles of microproteins discovered in the fruit fly underscore the fundamental role of this genetic model for understanding how microproteins influence animal development, physiology, and behavior.
